# Endoscopic treatment of anastomotic leakage after colorectal surgery by using polyglycolic acid sheets and fibrin glue

**DOI:** 10.1002/deo2.364

**Published:** 2024-04-09

**Authors:** Kurodo Koshino, Ryosuke Nakagawa, Kimitaka Tani, Hiroka Kondo, Fumi Maeda, Takeshi Ohki, Shimpei Ogawa, Shigeki Yamaguchi

**Affiliations:** ^1^ Department of Surgery Institute of Gastroenterology Tokyo Women's Medical University Tokyo Japan; ^2^ Department of Surgery Tokyo Metropolitan Tama‐Hokubu Medical Center Tokyo Japan

**Keywords:** anastomosis, colorectal surgery, fibrin glue, fistula, polyglycolic acid

## Abstract

We describe the case of a 66‐year‐old man with an anastomotic fistula after rectal surgery, which was treated colonoscopically using polyglycolic acid sheets and fibrin glue. Polyglycolic acid sheets and fibrin glue have been used in thoracic surgery and otolaryngology to reinforce sutures and prevent air leakage. There have been recent reports of their use in endoscopic surgery for the closure of intraoperative perforations after endoscopic submucosal dissection and for fistula closure after upper gastrointestinal tract surgery. However, anastomotic fistulas in colorectal surgery are difficult to visualize endoscopically and may be difficult to suture with clips due to fibrosis. Polyglycolic acid sheets can be easily trimmed, and the fistula can be easily filled using these sheets; moreover, using fibrin glue to fix the sheets may enable fistula closure in areas that are difficult to visualize endoscopically.

## INTRODUCTION

Gastrointestinal fistula formation can occur owing to perforation after endoscopic treatment or anastomotic leakage (AL) after surgery, or it can occur spontaneously because of persistent inflammation. Conservative treatment with antibiotics, fasting, and continuous aspiration with a drainage tube may sometimes improve symptoms. However, leakage of the intestinal fluid and fecal material into the abdominal cavity and abscess formation can be life‐threatening. With conservative management, such as drainage, recovery takes time, and surgical closure of the fistula is complicated by adhesions and fibrosis of the organ owing to inflammation.

Endoscopic closure of fistulas has usually been attempted using metal clips. Complete closure is difficult in some cases owing to the size of the fistula, fibrosis, and edema of the surrounding mucosa. In some cases, there may be stenosis and poor endoscopic operability and visual field, especially at anastomotic sites after gastrointestinal surgery.^1^ This is one factor that makes fistula closure difficult.

Bioabsorbable polyglycolic acid (PGA) sheets (Neoveil; Gunze Co.) have been used to reinforce sutures and prevent air leakage during surgery and have been recently used in endoscopic surgery to close intraoperative perforations,^2^ delayed perforations, and fistulas and to prevent postoperative bleeding and stenosis.^3^ Although there have been some reports of their use for the closure of anastomotic fistulas in the upper gastrointestinal tract,^4^, ^5^, ^6^ there have been no reports on using only PGA sheets and fibrin glue for the closure of anastomotic fistulas after colorectal surgery. Here, we describe a case in which PGA sheets and fibrin glue were used to close an anastomotic fistula after rectal surgery. We also examine the usefulness of this approach.

## CASE REPORT

The patient was a 66‐year‐old man with a diagnosis of rectal cancer diagnosis and a tailgut cyst who underwent robot‐assisted low anterior resection, covering ileostomy, and cyst resection in December 2019. Pathology results showed that he had rectal cancer pT3N0M0 pStage IIa (Japanese Society for Cancer of the Colon and Rectum, Ninth Edition) and an epidermoid cyst‐like lesion. Furthermore, the intraoperative leak test was positive; additional anastomosis suturing was performed and an intrapelvic drainage tube was placed. On the sixth postoperative day, the patient developed a fever, and computed tomography showed fluid retention in the pelvis. The patient developed sepsis; however, after continued drainage with an intrapelvic tube and antimicrobial treatment, he was discharged on postoperative day 39.

**FIGURE 1:**
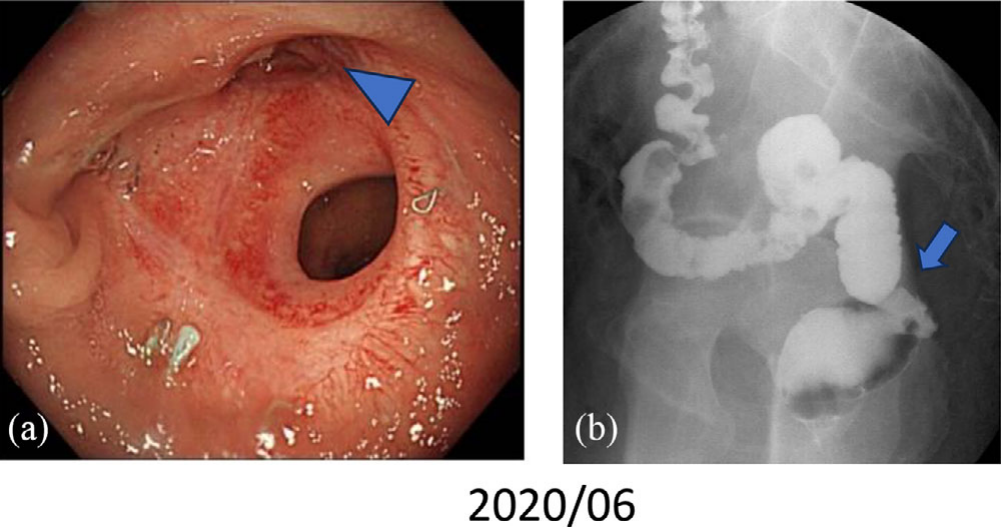
Colonoscopy and gastro‐enema: (a) Anastomotic stenosis and suspected fistula (arrowhead) in the rectum. (b) No leakage of the contrast medium from the anastomosis.

**FIGURE 2:**
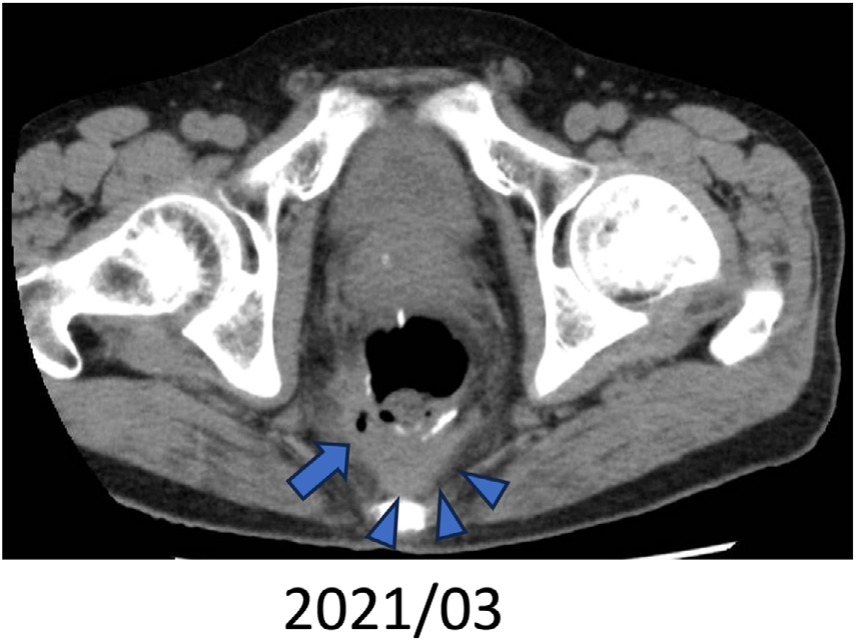
Image of computed tomography. Fluid retention on the dorsal side of the anastomosis.

**FIGURE 3:**
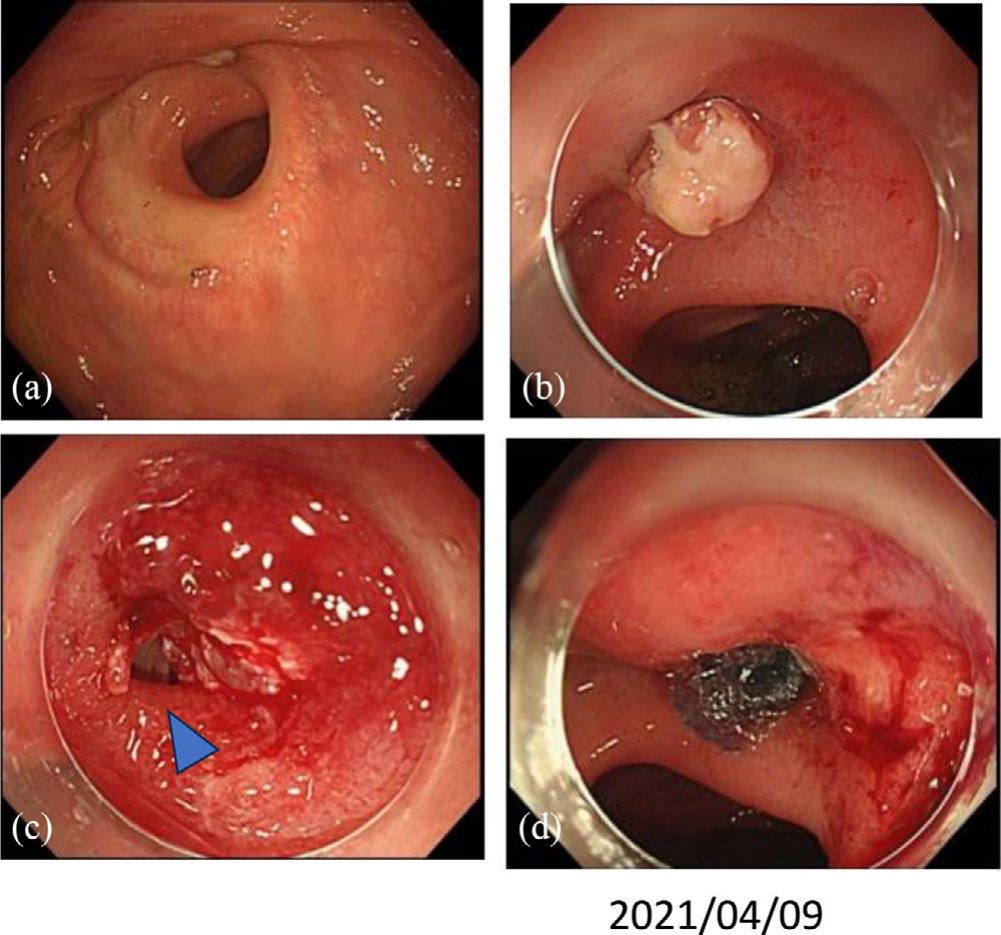
Endoscopic treatment of the anastomotic fistula: (a) Anastomosis and inflammatory polyp. (b) Anastomotic fistula after polypectomy (arrowhead). (c) Stuffing the polyglycolic acid sheet. (d) Spraying fibrin glue.

**FIGURE 4:**
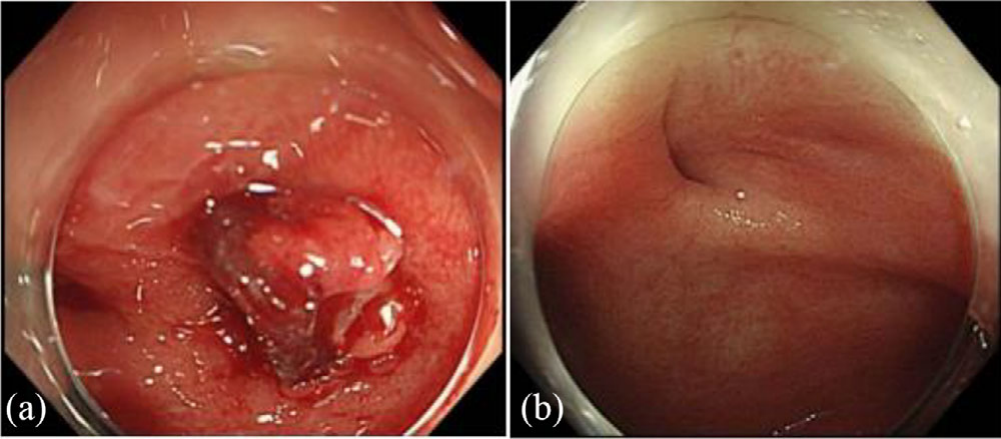
Endoscopy findings after treatment: (a) The fistula became small and tight approximately six months after endoscopic treatment. (b) The fistula was not found in the anastomosis approximately 1.5 years later.

Approximately 6 months after the surgery, we performed a colonoscopy (Figure 1a), which revealed a circumferential stenosis at the anastomosis and a suspected fistula in the posterior wall. An enema examination showed no extraintestinal leakage of the contrast medium (Figure 1b). Therefore, no fistula formation was diagnosed and stoma closure was performed. Approximately 1 year after the surgery, owing to discomfort in the perineum and purulent discharge during defecation, rectal AL was suspected. Computed tomography showed a small amount of fluid accumulation in the posterior wall of the pelvic anastomosis (Figure 2). Furthermore, colonoscopy revealed inflammatory granulation on the posterior wall of the anastomosis on the anorectal side (Figure 3a). Thus, an endoscopic polypectomy with a snare was performed, and a fistula was found at the same site (Figure 3b). Endoscopic closure was attempted; however, closure with a metal clip was considered difficult due to strong fibrosis. Therefore, PGA sheets and fibrin glue spray (Bolheal; Teijin Pharma Co.) were used for closure. The site was endoscopically cleaned with a spreading tube, and a 2‐cm^2^ PGA sheet was inserted. In the first step, biopsy forceps were inserted into the scope outside the body, and the PGA sheet was inserted between the forceps and stored in the hood. The endoscope was inserted transanally and delivered to the lesion. The procedure was then terminated by spreading fibrin glue, which was sprayed first with solution A (fibrinogen) and then with solution B (thrombin; Figure 3c,d). The same procedure was performed three times during a period of approximately 2 weeks. Six months later, this fistula was confirmed by colonoscopy. The same procedure was attempted; however, the fistula had become smaller and could no longer be adequately filled with PGA sheets; therefore, this treatment was terminated (Figure 4a). A colonoscopy performed approximately 1.5 years after the endoscopic treatment showed that the fistula had almost completely closed; furthermore, the patient's subjective symptoms had disappeared (Figure 4b).

## DISCUSSION

The treatment of AL after gastrointestinal resection is usually conservative treatment or re‐operation. Re‐operation for AL after colorectal surgery sometimes requires a colostomy, which may decrease the quality of life. Even in cases in which a diverting stoma has been created in advance, as in the present case, pelvic abscesses may develop due to AL. Therefore, there is an urgent need to establish a new treatment for AL.

The over‐the‐scope clip system has recently been reported as an alternative to clips for fistula closure. It has been used for perforation closure during endoscopic submucosal dissection. However, there are not many reports of its use for managing fistulas associated with postoperative AL in the gastrointestinal tract,^7^ and there have been reports of anastomosis narrowing due to inflammation and scarring of the surrounding tissue, which reduces the success rate.^8^, ^9^ Therefore, although the fistula was relatively small in our patient, the use of over‐the‐scope‐clip was deemed impossible because of the local scarring.

On the other hand, the PGA sheet, which is an absorbable non‐woven fabric sheet, has been used since the 1970s.^10^ It is used to reinforce the suture site and prevent air leakage.^2^ It is also a flexible material that can be easily trimmed to match the shape of the tissue, making it useful for preventing postoperative bleeding and scar stenosis after endoscopic submucosal dissection and other procedures.^7^ The combined use with fibrin glue forms a stable fibrin mass through the PGA sheet, which integrates with the sheet and allows long‐term adhesion of the PGA sheet even after the fibrin is dissolved, as the cellular and granulation components develop while surrounding the PGA fibers in the mass. This treatment with clips has been reported for AL after upper gastrointestinal surgery.^9^ However, there have been some reports of its use in combination with clips, and there have been no reports of this treatment alone being used for postoperative AL or fistula management after rectal surgery. In the present case, the fistula was relatively small, long, and thin, and it was considered possible that the PGA sheet remained in the fistula for a long time because it was trimmed and stuffed into the fistula, taking advantage of its characteristics, and spread with fibrin glue. However, there are limitations to this treatment. The use of PGA sheets and fibrin glue is also recommended for fistulas smaller than 2 cm.^6^ The size of this fistula was less than 2 cm, which was a good indication. Although there is no clear criterion for the necessity of intrapelvic fluid drainage, from the surgeon's point of view, careful follow‐up is considered a good indication for cases that are difficult to puncture by computed tomography or ultrasound in the absence of an elevated inflammatory response on laboratory data. In this case, the decision regarding the timing of stoma closure was also difficult, but if no fistula was evidenced by pressure on the enema test and there was no significant intestinal dilatation on the oral side above the anastomotic stenosis, this was sufficient reason to close the stoma.

In conclusion, closure of fistulas near the rigidly altered anastomotic site is difficult and requires a physician with advanced endoscopic skills to perform the clipping. On the other hand, in the present technique, no clips were used, and closure was possible using only PGA sheets and fibrin glue. The most important point of this report is that the device is simple to use and can be attempted even in facilities without highly specialized endoscopists.

Herein, we report a rare case of anastomotic fistula treatment. In the future, it may become a good treatment option for postoperative AL.

## CONFLICT OF INTEREST STATEMENT

None.
